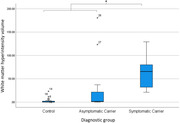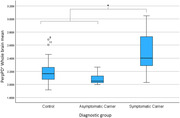# Detection of presymptomatic cerebral amyloid angiopathy, CAA, using diffusion MRI analysis of cortical grey matter in hereditary mutation carriers

**DOI:** 10.1002/alz.094145

**Published:** 2025-01-09

**Authors:** Steven A Chance, Ian Hardingham, Michele Valotti, Sanneke van Rooden, Jeroen van der Grond

**Affiliations:** ^1^ Oxford Brain Diagnostics, Oxford, Oxfordshire United Kingdom; ^2^ Oxford Brain Diagnostics, Oxford United Kingdom; ^3^ Leiden University Medical Center, Leiden Netherlands

## Abstract

**Background:**

HCHWA‐D mutation carriers experience highly heritable early onset of cerebral amyloid angiopathy (CAA). Diffusion MRI appears to be sensitive to CAA and detects punctate cortical lesions in CAA cases post‐mortem. DTI of white matter tracts was found to be sensitive to alterations in symptomatic HCHWA‐D carriers but did not show alterations in presymptomatic carriers (Schouten et al. https://doi.org/10.1161/JAHA.118.011288.). The diffusion signal from cortical grey matter microstructure may yet detect presymptomatic cases.

**Method:**

CDM (cortical disarray measurement) quantifies DTI parameters related to the vertical minicolumnar microstructure of cortical grey matter that is disrupted in neurodegeneration. AngleR is the angle between the principal diffusion direction and the cortical minicolumn axis. PerpPD+ and ParlPD are the components parallel and perendicular to the minicolumn axis. Participants from an existing dataset (Schouten et al.), included 12 symptomatic and 11 asymptomatic HCHWA‐D mutation carriers and 24 non‐carrier controls (based on: genotype, clinical status, and age). Diffusion MRI had been acquired at a single time point from a Philips Achieva 3T scanner at Leiden University Medical Center. Statistical GLM used multivariate ANOVA, classifying patients by Mutation status (carrier vs non‐carrier) and Clinical status (non‐carrier control, asymptomatic carrier, symptomatic carrier), covarying for age.

**Result:**

For the whole brain cortical average PerpPD+ was significantly increased in mutation carriers (p<0.05) driven by a significantly increased PerpPD+ in symptomatic carriers compared to both asymptomatic groups (controls and carriers) (p<0.05). At the regional level some measures in prespecified regions of interest were significantly altered in symptomatic carriers and, notably, significantly different in mutation carriers (p<0.05), both symptomatic and asymptomatic groups, compared to controls.

**Conclusion:**

Diffusion MRI analysis of cerebral cortex detects microstructural differences related to clinical symptoms of CAA in HCHWA‐D mutation carriers and particular measures of the microstructure organisation differentiate the CAA group, including presymptomatic, from controls.